# 4-Bromo­methyl-6-*tert*-butyl-2*H*-chromen-2-one

**DOI:** 10.1107/S1600536813015511

**Published:** 2013-06-08

**Authors:** H. Nagarajaiah, K. B. Puttaraju, K. Shivashankar, Noor Shahina Begum

**Affiliations:** a Department of Studies in Chemistry, Bangalore University, Bangalore 560 001, India

## Abstract

In the crystal structure of the title compound, C_14_H_15_BrO_2_, weak C—H⋯O inter­actions link the mol­ecules into zigzag chains extending along the *c*-axis direction. These chains are further assembled into (100) layers *via* π–π stacking inter­actions between inversion-related chromenone fragments [inter­planar distance = 3.376 (2) Å].

## Related literature

For therapeutic properties of coumarin derivatives, see: Lacy & O^’^Kennedy (2004 ▶); Mustafa *et al.* (2011 ▶). For structural features of coumarins, see: Moorthy *et al.* (2003 ▶). For related structures, see: Gowda *et al.* (2010 ▶); Fun *et al.* (2011 ▶).
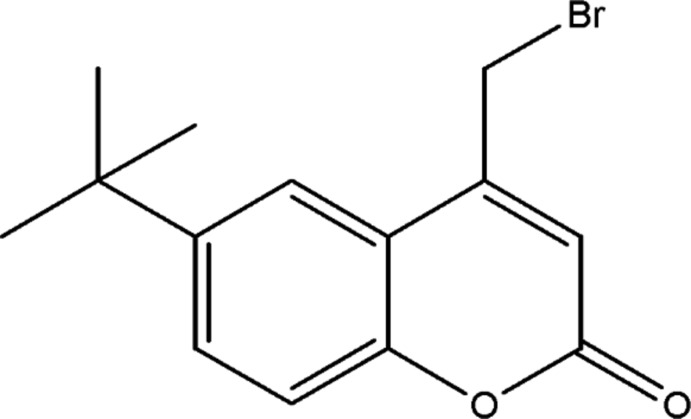


## Experimental

### 

#### Crystal data

C_14_H_15_BrO_2_
*M* *_r_* = 295.17
Monoclinic, 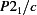

*a* = 10.3311 (19) Å
*b* = 16.830 (3) Å
*c* = 7.3374 (14) Å
β = 97.518 (3)°
*V* = 1264.8 (4) Å^3^
*Z* = 4
Mo *K*α radiation
μ = 3.24 mm^−1^
*T* = 100 K
0.18 × 0.16 × 0.16 mm

#### Data collection

Bruker SMART APEX CCD diffractometer
Absorption correction: multi-scan (*SADABS*; Bruker, 1998 ▶) *T*_min_ = 0.593, *T*_max_ = 0.625
7522 measured reflections
2737 independent reflections
2074 reflections with *I* > 2σ(*I*)
*R*_int_ = 0.040

#### Refinement

*R*[*F* ^2^ > 2σ(*F* ^2^)] = 0.041
*wR*(*F* ^2^) = 0.107
*S* = 1.05
2737 reflections
144 parameters
H-atom parameters constrained
Δρ_max_ = 0.78 e Å^−3^
Δρ_min_ = −0.36 e Å^−3^

### 

Data collection: *SMART* (Bruker,1998 ▶); cell refinement: *SAINT-Plus* (Bruker,1998 ▶); data reduction: *SAINT-Plus*; program(s) used to solve structure: *SHELXS97* (Sheldrick, 2008 ▶); program(s) used to refine structure: *SHELXL97* (Sheldrick, 2008 ▶); molecular graphics: *ORTEP-3 for Windows* (Farrugia, 2012 ▶) and *CAMERON* (Watkin *et al.*, 1996) ▶; software used to prepare material for publication: *WinGX* (Farrugia, 2012 ▶).

## Supplementary Material

Crystal structure: contains datablock(s) global, I. DOI: 10.1107/S1600536813015511/gk2575sup1.cif

Structure factors: contains datablock(s) I. DOI: 10.1107/S1600536813015511/gk2575Isup2.hkl

Supplementary material file. DOI: 10.1107/S1600536813015511/gk2575Isup3.cml

Additional supplementary materials:  crystallographic information; 3D view; checkCIF report

## Figures and Tables

**Table 1:** Hydrogen-bond geometry (Å, °)

*D*—H⋯*A*	*D*—H	H⋯*A*	*D*⋯*A*	*D*—H⋯*A*
C3—H3⋯O2^i^	0.95	2.42	3.334 (4)	162

Symmetry code: (i) 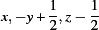.

**Fig. 1.**
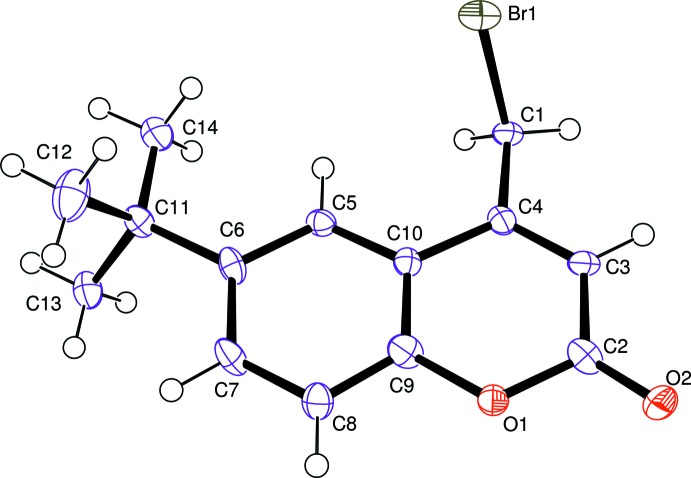
Molecular structure of the title compound showing 50% probability ellipsoids.

**Fig. 2.**
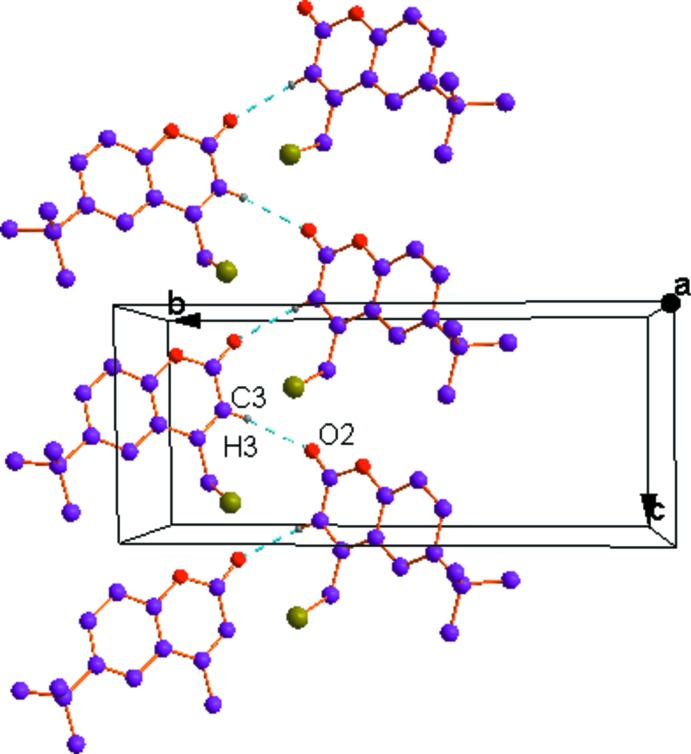
Chains of molecules formed by C-H···O interaction. Dotted lines indicate intermolecular interactions. H-atoms not involved in hydrogen bonding have been excluded.

## supplementary crystallographic information

### Comment

Coumarins are of great interest due to their biological properties (Lacy & O^'^Kennedy 2004). In particular, their physiological, bacteriostatic and anti-tumour activity (Mustafa *et al.*, 2011) makes these compounds attractive for further backbone derivatization and screening for their therapeutic properties.

In the title compound, C_15_H_14_BrO_2 _(Fig. 1), the coumarin ring is substituted with bromomethyl group at C4 and *tert*-butyl group at C6. The coumarin ring is essentialy planar (r.m.s. deviation = 0.019 Å). Among the three methyl groups belonging to *tert*-butyl moiety two methyl groups, C12 & C13, deviate from the plane of the coumarin ring whereas the carbon atom C14 of the methyl group lies within the plane. The crystal structure is stabilized by C—H···O interactions (Moorthy *et al.* 2003). The C3—H3···O2 interaction results in zigzag chains running along the *c*-axis (Fig. 2). There are intermolecular π–π interactions between two anti-parallel molecules in the unit cell with an interplanar distance of 3.376 (2) Å. For crystal structures related to the title compound, see: Gowda *et al.* (2010); Fun *et al.* (2011).

### Experimental

To a mixture of equimolar quantity of 4-*tert*-butyl phenol (0.1 mol) and 4-bromoethyl acetoacetate (0.1 mol) was added dropwise Conc. sulfuric acid (30 ml) with constant stirring and maintaining the temperature between 273–278 K. The reaction mixture was allowed to stand in ice chest overnight and deep red coloured solution was poured into the stream of crushed ice. Solid separated was filtered and washed with water and then with cold ethanol so as to get a colourless compound. Finally, it was recrystallized from ethyl acetate. Yield 89%; colorless solid; m.p. 417–420 K; IR (KBr, cm^-^1): 1700 (lactone C═ O), ^1^H NMR (300 MHz, DMSO-d_6_): δ 1.32 (s, 9H, 6-*tert*-butyl), 4.93 (s, 2H, CH2–Br), 6.70 (s, 1H, C3–H), 7.34 (d, 1H, C7–H, *J* = 6.2 Hz), 7.68 (d, 1H, C8–H, *J* = 8.1 Hz), 7.80 (s, 1H, C5–H): LC—MS 297 [*M* + 2]: Anal. Cald. for C_15_H_14_Br_1_O_2_: C 56.97; H 5.12. Found: C 56.91; H 5.04.

### Refinement

The H atoms were placed at calculated positions in the riding model approximation with C–H = 0.95, 0.98, and 0.99 Å for aryl, methyl, and methylene H-atoms respectively, with *U*_iso_(H) = 1.5*U*_eq_(C) for methyl H atoms and *U*_iso_(H) = 1.2*U*_eq_(C) for other H atom.

### Crystal data

**Table:**

C_14_H_15_BrO_2_	*F*(000) = 600
*M**_r_* = 295.17	*D*_x_ = 1.550 Mg m^−^^3^
Monoclinic, *P*2_1_/*c*	Mo *K*α radiation, λ = 0.71073 Å
Hall symbol: -P 2ybc	Cell parameters from 2074 reflections
*a* = 10.3311 (19) Å	θ = 2.3–27.0°
*b* = 16.830 (3) Å	µ = 3.24 mm^−^^1^
*c* = 7.3374 (14) Å	*T* = 100 K
β = 97.518 (3)°	Block, colourless
*V* = 1264.8 (4) Å^3^	0.18 × 0.16 × 0.16 mm
*Z* = 4	

### Data collection

**Table:**

Bruker SMART APEX CCD diffractometer	2737 independent reflections
Radiation source: fine-focus sealed tube	2074 reflections with *I* > 2σ(*I*)
Graphite monochromator	*R*_int_ = 0.040
ω scans	θ_max_ = 27.0°, θ_min_ = 2.3°
Absorption correction: multi-scan (*SADABS*; Bruker, 1998)	*h* = −13→12
*T*_min_ = 0.593, *T*_max_ = 0.625	*k* = −21→16
7522 measured reflections	*l* = −9→9

### Refinement

**Table:**

Refinement on *F*^2^	Primary atom site location: structure-invariant direct methods
Least-squares matrix: full	Secondary atom site location: difference Fourier map
*R*[*F*^2^ > 2σ(*F*^2^)] = 0.041	Hydrogen site location: inferred from neighbouring sites
*wR*(*F*^2^) = 0.107	H-atom parameters constrained
*S* = 1.05	*w* = 1/[σ^2^(*F*_o_^2^) + (0.0531*P*)^2^] where *P* = (*F*_o_^2^ + 2*F*_c_^2^)/3
2737 reflections	(Δ/σ)_max_ < 0.001
144 parameters	Δρ_max_ = 0.78 e Å^−^^3^
0 restraints	Δρ_min_ = −0.36 e Å^−^^3^

### Special details

**Table:**

Geometry. All esds (except the esd in the dihedral angle between two l.s. planes) are estimated using the full covariance matrix. The cell esds are taken into account individually in the estimation of esds in distances, angles and torsion angles; correlations between esds in cell parameters are only used when they are defined by crystal symmetry. An approximate (isotropic) treatment of cell esds is used for estimating esds involving l.s. planes.
Refinement. Refinement of *F*^2^ against ALL reflections. The weighted *R*-factor *wR* and goodness of fit *S* are based on *F*^2^, conventional *R*-factors *R* are based on *F*, with *F* set to zero for negative *F*^2^. The threshold expression of *F*^2^ > 2σ(*F*^2^) is used only for calculating *R*-factors(gt) *etc*. and is not relevant to the choice of reflections for refinement. *R*-factors based on *F*^2^ are statistically about twice as large as those based on *F*, and *R*- factors based on ALL data will be even larger.

### Fractional atomic coordinates and isotropic or equivalent isotropic displacement parameters (Å^2^)

**Table:**

	*x*	*y*	*z*	*U*_iso_*/*U*_eq_	
C1	0.4032 (3)	0.13349 (18)	0.2373 (4)	0.0198 (7)	
H1A	0.4081	0.0849	0.1624	0.024*	
H1B	0.4768	0.1686	0.2164	0.024*	
C2	0.5105 (3)	0.13683 (17)	0.7557 (4)	0.0190 (6)	
C3	0.4913 (3)	0.15577 (18)	0.5616 (4)	0.0190 (6)	
H3	0.5340	0.2011	0.5205	0.023*	
C4	0.4148 (3)	0.11146 (17)	0.4361 (4)	0.0173 (6)	
C5	0.2620 (3)	−0.00601 (18)	0.3828 (4)	0.0171 (6)	
H5	0.2467	0.0057	0.2551	0.021*	
C6	0.1996 (3)	−0.07068 (17)	0.4490 (4)	0.0187 (6)	
C7	0.2270 (3)	−0.08765 (18)	0.6383 (4)	0.0215 (7)	
H7	0.1870	−0.1324	0.6869	0.026*	
C8	0.3104 (3)	−0.04076 (18)	0.7543 (4)	0.0215 (7)	
H8	0.3276	−0.0530	0.8816	0.026*	
C9	0.3687 (3)	0.02395 (17)	0.6843 (4)	0.0182 (6)	
C10	0.3472 (3)	0.04295 (17)	0.4984 (4)	0.0161 (6)	
C11	0.1007 (3)	−0.12192 (18)	0.3272 (4)	0.0210 (7)	
C12	−0.0343 (3)	−0.11188 (14)	0.3930 (5)	0.0368 (9)	
H12A	−0.0993	−0.1429	0.3137	0.055*	
H12B	−0.0590	−0.0556	0.3871	0.055*	
H12C	−0.0301	−0.1307	0.5200	0.055*	
C13	0.1402 (3)	−0.21004 (14)	0.3427 (4)	0.0265 (7)	
H13A	0.2251	−0.2171	0.2987	0.040*	
H13B	0.0744	−0.2421	0.2680	0.040*	
H13C	0.1465	−0.2269	0.4716	0.040*	
C14	0.0902 (2)	−0.09867 (5)	0.12412 (5)	0.0305 (8)	
H14A	0.1753	−0.1056	0.0808	0.046*	
H14B	0.0632	−0.0430	0.1096	0.046*	
H14C	0.0255	−0.1326	0.0520	0.046*	
O1	0.45022 (6)	0.06915 (5)	0.80902 (6)	0.0197 (5)	
O2	0.57521 (5)	0.17418 (5)	0.87563 (5)	0.0241 (5)	
Br1	0.23783 (3)	0.18813 (2)	0.16045 (4)	0.03299 (15)	

### Atomic displacement parameters (Å^2^)

**Table:**

	*U*^11^	*U*^22^	*U*^33^	*U*^12^	*U*^13^	*U*^23^
C1	0.0208 (16)	0.0200 (16)	0.0194 (15)	0.0001 (12)	0.0053 (12)	0.0043 (12)
C2	0.0205 (16)	0.0150 (15)	0.0227 (16)	0.0037 (12)	0.0073 (13)	−0.0024 (12)
C3	0.0178 (16)	0.0190 (16)	0.0205 (15)	0.0001 (12)	0.0036 (12)	0.0029 (12)
C4	0.0161 (15)	0.0153 (15)	0.0208 (15)	0.0031 (12)	0.0035 (12)	0.0013 (12)
C5	0.0165 (15)	0.0185 (16)	0.0164 (14)	0.0038 (12)	0.0018 (12)	0.0026 (11)
C6	0.0170 (15)	0.0157 (15)	0.0246 (16)	0.0017 (12)	0.0066 (12)	−0.0007 (12)
C7	0.0251 (17)	0.0155 (15)	0.0252 (16)	0.0007 (13)	0.0082 (13)	0.0021 (12)
C8	0.0257 (17)	0.0232 (17)	0.0164 (15)	0.0013 (13)	0.0063 (13)	0.0009 (12)
C9	0.0189 (16)	0.0202 (16)	0.0159 (14)	0.0016 (12)	0.0036 (12)	−0.0034 (12)
C10	0.0163 (15)	0.0146 (14)	0.0177 (14)	0.0035 (11)	0.0038 (11)	−0.0021 (11)
C11	0.0219 (16)	0.0153 (15)	0.0260 (17)	−0.0002 (12)	0.0046 (13)	0.0010 (13)
C12	0.0241 (19)	0.030 (2)	0.057 (2)	−0.0062 (15)	0.0107 (17)	−0.0144 (18)
C13	0.034 (2)	0.0228 (17)	0.0230 (17)	−0.0016 (14)	0.0032 (14)	−0.0003 (13)
C14	0.033 (2)	0.0261 (19)	0.0288 (18)	−0.0086 (15)	−0.0094 (15)	0.0047 (14)
O1	0.0252 (12)	0.0186 (11)	0.0149 (10)	−0.0026 (9)	0.0014 (9)	0.0002 (8)
O2	0.0283 (13)	0.0232 (12)	0.0202 (11)	−0.0030 (9)	0.0010 (9)	−0.0037 (9)
Br1	0.0305 (2)	0.0332 (2)	0.0326 (2)	0.00355 (15)	−0.00600 (15)	0.01003 (15)

### Geometric parameters (Å, º)

**Table:**

C1—C4	1.494 (4)	C8—C9	1.376 (4)
C1—Br1	1.957 (3)	C8—H8	0.9500
C1—H1A	0.9900	C9—O1	1.387 (3)
C1—H1B	0.9900	C9—C10	1.390 (4)
C2—O2	1.209 (3)	C11—C14	1.531 (3)
C2—O1	1.379 (3)	C11—C13	1.539 (4)
C2—C3	1.448 (4)	C11—C12	1.544 (4)
C3—C4	1.356 (4)	C12—H12A	0.9800
C3—H3	0.9500	C12—H12B	0.9800
C4—C10	1.452 (4)	C12—H12C	0.9800
C5—C6	1.385 (4)	C13—H13A	0.9800
C5—C10	1.407 (4)	C13—H13B	0.9782
C5—H5	0.9500	C13—H13C	0.9819
C6—C7	1.410 (4)	C14—H14A	0.9800
C6—C11	1.532 (4)	C14—H14B	0.9800
C7—C8	1.378 (4)	C14—H14C	0.9800
C7—H7	0.9500		
			
C4—C1—Br1	110.7 (2)	O1—C9—C10	121.8 (3)
C4—C1—H1A	109.5	C9—C10—C5	117.6 (3)
Br1—C1—H1A	109.5	C9—C10—C4	118.0 (3)
C4—C1—H1B	109.5	C5—C10—C4	124.3 (3)
Br1—C1—H1B	109.5	C14—C11—C6	112.4 (2)
H1A—C1—H1B	108.1	C14—C11—C13	107.6 (2)
O2—C2—O1	116.8 (2)	C6—C11—C13	110.4 (2)
O2—C2—C3	126.4 (3)	C14—C11—C12	109.0 (2)
O1—C2—C3	116.9 (2)	C6—C11—C12	108.4 (2)
C4—C3—C2	122.7 (3)	C13—C11—C12	108.9 (2)
C4—C3—H3	118.7	C11—C12—H12A	109.5
C2—C3—H3	118.7	C11—C12—H12B	109.5
C3—C4—C10	119.0 (3)	H12A—C12—H12B	109.5
C3—C4—C1	119.4 (3)	C11—C12—H12C	109.5
C10—C4—C1	121.6 (3)	H12A—C12—H12C	109.5
C6—C5—C10	122.1 (3)	H12B—C12—H12C	109.5
C6—C5—H5	118.9	C11—C13—H13A	109.5
C10—C5—H5	118.9	C11—C13—H13B	109.4
C5—C6—C7	117.6 (3)	H13A—C13—H13B	109.5
C5—C6—C11	122.9 (3)	C11—C13—H13C	109.5
C7—C6—C11	119.6 (3)	H13A—C13—H13C	109.4
C8—C7—C6	121.4 (3)	H13B—C13—H13C	109.5
C8—C7—H7	119.3	C11—C14—H14A	109.5
C6—C7—H7	119.3	C11—C14—H14B	109.5
C9—C8—C7	119.4 (3)	H14A—C14—H14B	109.5
C9—C8—H8	120.3	C11—C14—H14C	109.5
C7—C8—H8	120.3	H14A—C14—H14C	109.5
C8—C9—O1	116.4 (2)	H14B—C14—H14C	109.5
C8—C9—C10	121.8 (3)	C2—O1—C9	121.59 (17)
			
O2—C2—C3—C4	−178.6 (3)	C6—C5—C10—C9	0.6 (4)
O1—C2—C3—C4	2.0 (4)	C6—C5—C10—C4	−179.9 (3)
C2—C3—C4—C10	1.1 (4)	C3—C4—C10—C9	−2.7 (4)
C2—C3—C4—C1	−178.1 (3)	C1—C4—C10—C9	176.5 (3)
Br1—C1—C4—C3	−102.3 (3)	C3—C4—C10—C5	177.8 (3)
Br1—C1—C4—C10	78.5 (3)	C1—C4—C10—C5	−3.0 (4)
C10—C5—C6—C7	−1.6 (4)	C5—C6—C11—C14	5.8 (4)
C10—C5—C6—C11	176.5 (3)	C7—C6—C11—C14	−176.1 (3)
C5—C6—C7—C8	1.4 (4)	C5—C6—C11—C13	126.0 (3)
C11—C6—C7—C8	−176.8 (3)	C7—C6—C11—C13	−55.9 (3)
C6—C7—C8—C9	−0.2 (5)	C5—C6—C11—C12	−114.7 (3)
C7—C8—C9—O1	179.2 (2)	C7—C6—C11—C12	63.4 (3)
C7—C8—C9—C10	−0.9 (5)	O2—C2—O1—C9	176.86 (19)
C8—C9—C10—C5	0.7 (4)	C3—C2—O1—C9	−3.7 (3)
O1—C9—C10—C5	−179.4 (2)	C8—C9—O1—C2	−177.8 (2)
C8—C9—C10—C4	−178.9 (3)	C10—C9—O1—C2	2.3 (4)
O1—C9—C10—C4	1.0 (4)		

### Hydrogen-bond geometry (Å, º)

**Table:**

*D*—H···*A*	*D*—H	H···*A*	*D*···*A*	*D*—H···*A*
C3—H3···O2^i^	0.95	2.42	3.334 (4)	162

Symmetry code: (i) *x*, −*y*+1/2, *z*−1/2.
